# Oswald Schmiedeberg (1838–1921): Ninth Pharmacologic‑Historical Forum, 2024, Munich, Germany

**DOI:** 10.1007/s00210-024-03306-1

**Published:** 2024-07-23

**Authors:** Helmut Greim

**Affiliations:** Technical University of Munich, Munich, Germany

**Keywords:** Dorpat, Strasbourg, Pharmacology, John Jacob Abel, Rudolf Buchheim, Arthur Robertson Cushny, Wofgang Heubner, Otto Loewi, Hans Horst Meyer, Bernhard Naunyn, Albert Schweizer, George Wallace

## Abstract

Oswald Schmiedeberg was born in one of the former Baltic provinces of Russia. He studied medicine in Dorpat (Tartu) and joined the Institute of Pharmacology of Rudolf Buchheim in Dorpat. After promotion (1866) and habilitation (1868), he succeeded Buchheim as director of the institute. During this time, he further developed the experimental methods leading to the improvement of pharmacological knowledge introduced by Buchheim. In 1872, he became director of the Institute of Pharmakologie of the newly founded Kaiser-Wilhelm University in Strasbourg. He held this position for over 42 years until the end of the World War 1 when all Germans had to leave the former Reichsland Elsass-Lothringen. He settled next to his friend and colleague Naunyn in Baden-Baden, where he died in 1921. Holmstedt and Liljestrand’s (1963) History of Pharmacology and Toxicology noted, “Schmiedeberg was undoubtedly the most prominent pharmacologist of his time.” He had about 120 pupils, about 40 of them occupied pharmacology chairs throughout the world. In the USA, John Jacob Abel, after his return to the USA, became the “father of American pharmacology”. In 1873, Schmiedeberg, together with the pathologist Klebs (Prague) and the clinician Naunyn (Königsberg), founded the Archiv für experimentelle Pathologie und Pharmakologie. When Naunyn died in 1925, the periodical was named Naunyn–Schmiedeberg’s Archiv, from volume 110 onwards. In 1969, the designation “experimental pathology” was dropped, since nearly all papers submitted for some time past dealt with pharmacology. In 1883, Schmiedeberg published the *Grundriss der Arzneimittellehre*, the later edits with the title *Grundriss der Pharmakologie in Bezug auf Arzneimittellehre und Toxikologie*.

## Oswald Schmiedeberg

**Fig. 1:**
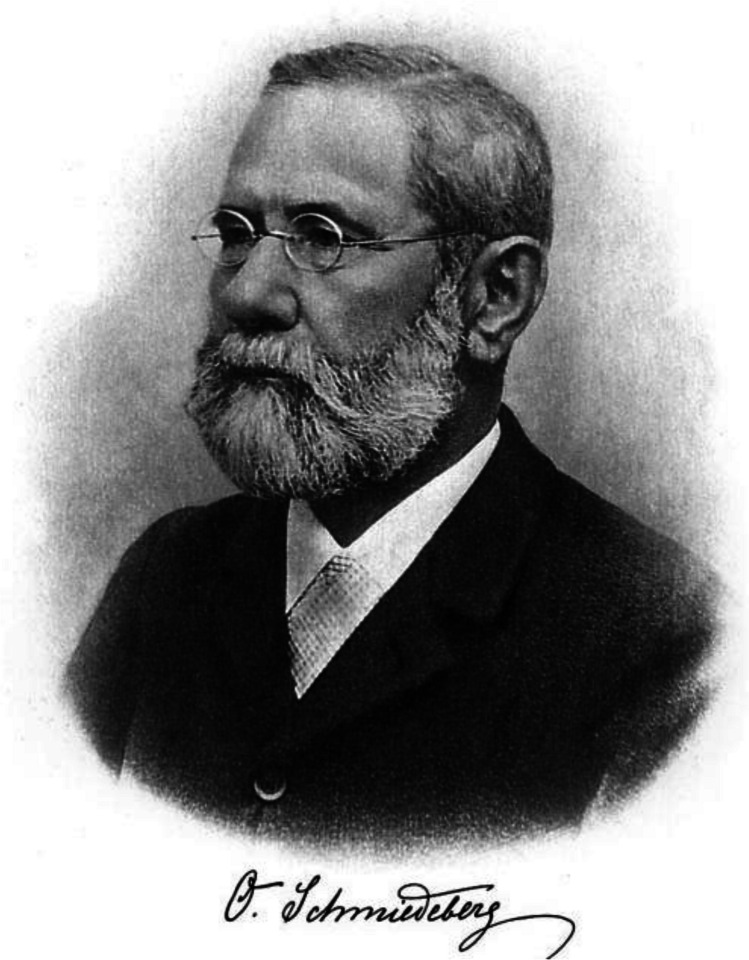
Oswald Schmiedeberg 1908 (Archive of DGPT)

Oswald Schmiedeberg was born September 29, 1838, as a son of a forester in a small place in Courland, one of the former Baltic provinces of Russia (Fig. 1). After visiting the Gymnasium in Dorpat (today Tartu), he studied medicine at the University of Dorpat between 1860 and 1866. During this time, he became interested in pharmacology and became a student of Buchheim at the Institute of Pharmacology. After completing his MD thesis (quantitative measurement and fate of chloroform in the blood), he joined the institute as a scientific assistant. In 1867, he became Privat Dozent, and in 1969, an extraordinary professor. During 1869, he visited the physiologist Carl Ludwig at the University of Leipzig to acquire specific experimental tools like the Kymograph and the isolated frog heart, which have not been available in Dorpat. When Buchheim moved to the University Gießen in 1871, he was appointed as his successor in Dorpat.

Buchheim when studying medicine at the University of Leipzig translated Jonathan Pereira’s “The Elements of Materia Medica and Therapeutics.” Buchheim revised its content, omitted drugs which he considered to be ineffective, replaced them by others, and tried to give a rationale for therapy. Last but not least in his experimental work for his MD thesis at the Institute of the Physiologist Ernst-Heinrich Weber in Leipzig, he realized that there is an urgent need to justify the use of drugs by experimental studies and added a specific chapter “Mode of Action” on drugs in which he described those drugs which have been investigated experimentally to understand the pharmacological effects.

This task took him 4 years, and in addition, other editorial work and publications made him well known, and on the recommendation of the physiologist Friedrich Bidder, dean of the faculty, 2 years after completing his MD thesis, he became chair of Materia Medica, Dietetics and History, and *Encyclopedia of Medicine* at the University of Dorpat in 1847. There he started his experimental pharmacological work in the basement of his own house and, in 1860, moved into the newly built Institute of Pharmacology with sufficient facilities for experimental research (Muscholl 1995; Philippou and Seifert 2022).

Buchheim has not achieved a specific major discovery, but his introduction of experimental studies to explore the effects of compounds led to an improvement in the understanding of pharmacological effects.

After Buchheim left for Gießen, Schmiedeberg continued the experimental research. His major achievement between 1871 and 1872 has been the identification of *muscarine* as the *stimulant* of the nervus *vagus* and its antidote atropine. This work published with Koppe presented for the first time a specific antidote, which has been evaluated in animal experiments suitable for clinical application.

During these years, Bernhard Naunyn (1839–1925) moved from Berlin to Dorpat as director of the university clinic of internal medicine, which was the beginning of a life-long cooperation and friendship between Naunyn and Schmiedeberg.

In 1872, Schmiedeberg became director of the Institute of *Pharmacology* of the newly founded Kaiser-Wilhelm University in Strasbourg, which after the French-German war 1870/1871 has been founded in the newly established German province Elsass-Lothringen. It was well equipped with financial resources and had the opportunity to recruit promising scientists from the German-speaking parts of Europe.

Together with Schmiedeberg, other renown professors have been appointed: the professor of anatomy Heinrich Wilhelm Waldeyer (1845–1921), who described the lymphatic Rachenring; the physiologist Friedrich Goltz (1834–1902), Felix Hoppe-Seyler (1825–1895), one of the founders of biochemistry and the *Zeitschrift für physiologische Chemie*, later *Hoppe-Seylers Zeitschrift für physiologische Chemie*; and the pathologist Friedrich Daniel von Recklinghausen (1833–1910), who described the osteodystrophia fibrosa generalisata cystica, a consequence of hyperparathyreoidism and the neurofibromatosis type 1. In 1888, Naunyn joined from Königsberg, and both friends cooperated in Strasbourg until Naunyn retired in 1904 and moved to Baden-Baden.

Schmiedeberg was one of the youngest of these in 1872 appointed professors and held this position for over 42 years until 1919, when all in Elsass-Lothringen living Germans had to leave. At that time, he was the last of the 1872 appointed professors of the medical faculty of the Kaiser-Wilhelm Universität.

During the first years in Strasbourg, Schmiedeberg’s experimental facilities in the former Faculté de Médicine have been limited until he moved in 1887 to the spacious new institute, which he had planned together with the architect Otto Warth (1845–1918).

Research in Strasbourg included hypnotic effects of urea derivatives and paraldehyde, the action of digitalis on the heart muscle, nicotine as blocker of cardiac vagal ganglia, central and peripheral effects of caffeine, toxicity of heavy metals and their organic complexes, formation of hippuric acid in the kidney and of urea in the liver, detoxication of various organic compounds by forming conjugates with glucuronic acid, and toxicity of muscarin. For more details, see Schmiedeberg (1870, 1872, 1886), Bunge and Schmiedeberg (1877), Schmiedeberg and Koppe (1869), and Schmiedeberg and Meyer (1879).

During his time in Strasbourg, Schmiedeberg had about 120 pupils from 20 different countries. Because his advice to faculties was highly appreciated, he helped many of them to acquire good positions. About 40 of them occupied pharmacology chairs throughout the world (Koch-Weser and Schechter 1978). For a comprehensive representation of all Schmiedeberg’s scholars in Strasbourg, see Philippou and Seifert (2022).

**Fig. 2:**
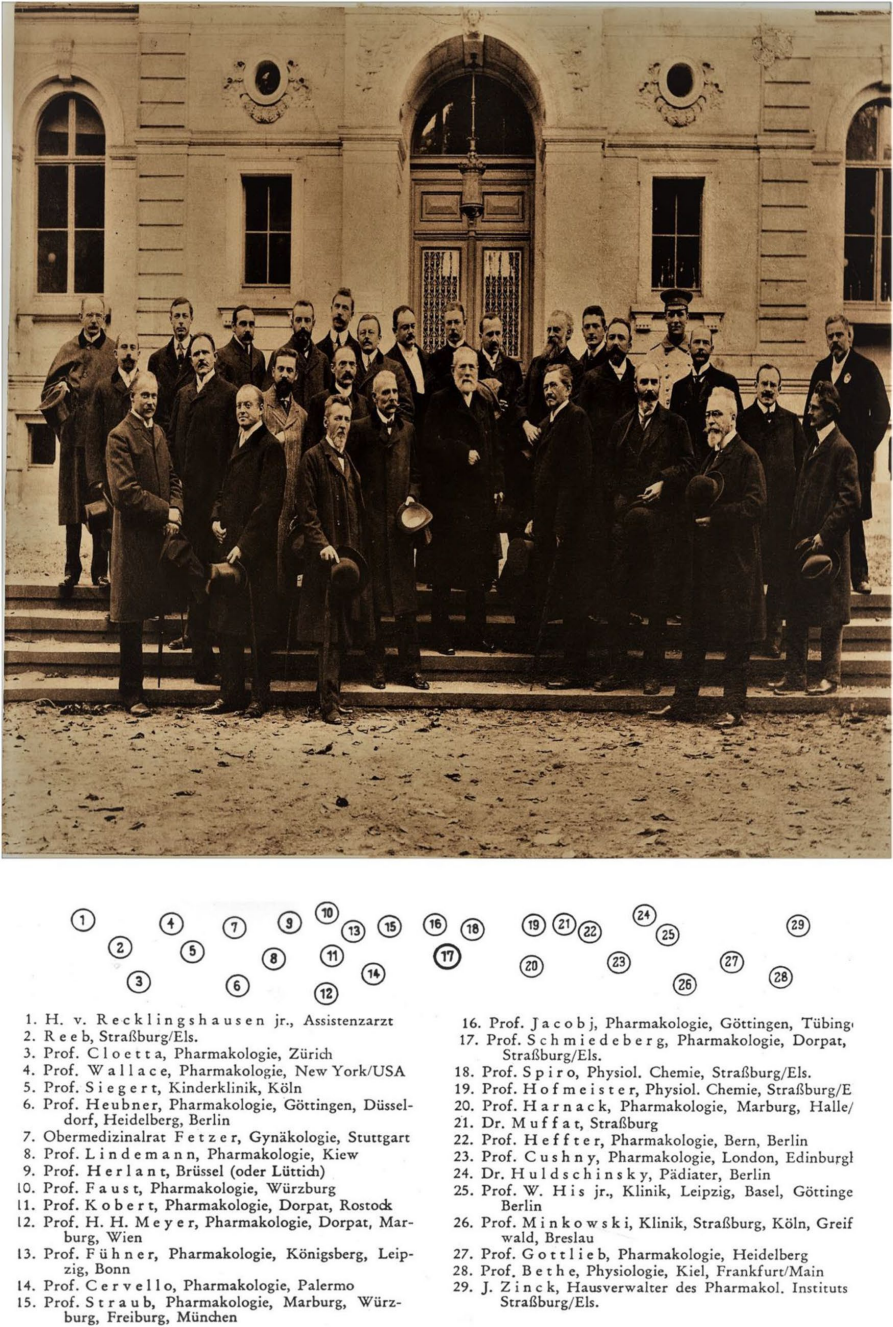
Schmiedeberg at his 70th birthday (October 11, 1908) together with his pupils and friends in Strasbourg (Archive of DGPT)

On the occasion of his 70th birthday in 1908, many of his previous pupils, who are presented in the group picture, assembled at Strasbourg (Fig. 2). Apart from German pharmacologists like Wolfgang Heubner (at that time in Göttingen, later in Heidelberg and Berlin) and clinicians like Oscar Mikowski (professor of medicine in Breslau), many nationalities had come to Strasbourg, as the professors of Pharmacology Cloetta (Zurich), Wallace (New York), Lindemann (Kiev), Herlant (toxicologist at Brussels), Cervello (Palermo), Hofmeister (Prague), Kobert (Dorpat and Rostock), Cushny (at that time in London), and Hans Horst Meyer (Vienna) (Philippou and Seifert 2022).

A prominent American pharmacologist who worked in Schmiedebergs laboratory spent 7 years in Germany to complete his pharmacological education was John Jacob Abel (1857–1938). He was born in Ohio and also worked with Ludwig and Boehm. Returning to America in 1891 on the recommendation of Schmiedeberg, he became the first professor of pharmacology at Ann Arbor (University of Michigan) and moved to Johns Hopkins 2 years later. Working in the laboratory until the age of 80, he had an enormous methodological spectrum and experience, which he conferred to his pupils. His major work has been the purification of adrenaline, pituitary hormones, and insulin. In 1909, Abel, 36 years after the Archiv für experimentelle Pathologie und Pharmakologie, founded the Journal of Pharmacology and Experimental Therapeutics. He has been called the father of American pharmacology.

Abel’s successor in Ann Arbor has been Arthur Robertson Cushny (1866–1926). Born at Fochabers near Elgin, Scottland, he received his medical education in Aberdeen. With a Thompson Fellowship, he went to Bern for a year to be trained in physiology by Kronecker (1839–1914). Then, he entered Schmiedeberg’s department, where after 1 year, he was appointed to one of the precious assistant positions. After 3 years in Strasbourg and on Schmiedeberg’s recommendation, he became the successor of Abel at the pharmacology chair at Ann Arbor. After 12 years, he returned to Britain to occupy the newly created chair at University College, London, and in 1920, he went to Edinburgh. He is known for his digitalis studies and for his equally famous work on kidney functions. Cushny published an extremely successful Textbook of Pharmacology which, after eight editions in his lifetime, was continued by later editors.

Hans Horst Meyer (1853–1939) may have had the greatest impact on pharmacology of Schmiedeberg’s pupils. He was born in Insterburg, East Prussia, studied medicine in Königsberg, Leipzig, and Berlin, and after his promotion to Doctor of Medicine in Königsberg, he joined Schmiedeberg’s institute in Strasbourg. In 1881, he was appointed to the chair of pharmacology in Dorpat, went to Marburg, and finally to Vienna where he stayed until his death. Together with Schmiedeberg, he discovered glucuronic acid as the most important reaction partner of drugs. He also described the relationship between the lipophilicity of general anesthetics and their potency. A year later, Charles Ernest Overton (1865–1933) came to the same conclusion, and this correlation became known as the Meyer-Overton hypothesis. Meyer was also one of the founding members of the German Pharmacological Society in 1920. Four of his pupils won the Nobel Prize: George Hoyt Whipple (1934), Otto Loewi (1936), Corneille Heymans (1938), and Carl Ferdinand Cori (1947). Schmiedeberg himself has been nominated repeatedly (Pohar and Hansson 2020).

One of his pupils, Otto Loewi (1873–1961), spent 11 years with Meyer, 7 in Marburg, and 4 in Vienna, before he accepted the chair of pharmacology at Graz University. Being Jewish, after the German Anschluss, he was forced to leave Austria and eventually immigrated in 1940 to the USA, where he had a position as a professor of pharmacology at the New York University. There he worked with George Wallace, a former pupil of Schmiedeberg.

Visiting the Institute of Pharmacology at the University of Graz, I found a note of Albert Einstein, living in Princeton, to Otto Loewi’s 80th birthday in 1953, which he celebrated in New York City^1^:

**Table:** 

Heute wünschen wir, dass Du froh und in Seelenruhe diesen Tag begehst und die künftigen Denn man lernt in der langen Zeit, sich nicht mehr zu giften über die Thorheiten der Anderen, und sich mit Humor abzufinden mit den eigenen	Today we wish you to celebrate, happy and peaceful, This and many upcoming days, Because one learns over the long journey Not to be bothered by alien foolishness And with humor to tolerate our own

In 1873, Schmiedeberg, together with the pathologist Klebs (Prague) and the clinician Naunyn (Königsberg), founded the Archiv für experimentelle Pathologie und Pharmakologie. When Naunyn died in 1925, the periodical was named Naunyn–Schmiedeberg’s Archiv, from volume 110 onwards. In 1969, the designation “experimental pathology” was dropped, since nearly all papers submitted dealt with pharmacology.

In 1883, Schmiedeberg published the *Grundriss der Arzneimittellehre*, later edits had the title *Grundriss der Pharmakologie in Bezug auf Arzneimittellehre und Toxikologie* (Schmiedeberg 1906), and in 1912, the book *Arzneimittel und Genußmittel* (Schmiedeberg 1912).

Holmstedt and Liljestrand’s (1963) History of Pharmacology and Toxicology noted, “Schmiedeberg was undoubtedly the most prominent pharmacologist of his time.” He had about 120 pupils, about 40 of them occupied pharmacology chairs throughout the world. In the USA, John Jacob Abel, after his return to the USA, became the “father of American pharmacology.” For more details of Schmiedeberg’s life and achievements, see Meyer (1922), Koch-Weser and Schechter (1978), Muscholl (1995), and Philippou and Seifert (2022).

Wolfgang Heubner, who worked in the Strasbourg Institute between 1903 and 1908, published an interesting description of Schmiedeberg’s persona (Heubner 1956) of which parts are presented:

To me his persona was imposing and absolutely original. He was of medium stature, had full grey hair, a roundly cut full beard and a rather broad round nose. His gait was like heaving, much as that of a mariner’s at seaside. His speech had an unsullied Baltic accent, refreshed yearly during his summer vacations. For my eight years with him, Schmiedeberg’s clothing remained the same; he wore a grey suit with long coattails and a flat grey cravat around his white turn-down collar. His hat was a black Italian ‘Borsalino’ with a rather broad brim. Before wearing it, he pressed a longitudinal fold along, but after putting it on his head he pulled it down with both hands so that the nice fold bulged up and this hat enthroned more like a pot on his crown. He made extensive use of his university vacations. In early March he always went to Italy, returning by end of April. Early in August he travelled to his Baltic homeland, showing up in Strasbourg only by the end of October. During nice days at his Baltic home he went fishing or hunting, while on bad weather he sat at his desk finding pleasure and concentration in his scientific work.

In 1919, all Germans living in Elsass-Lothringen had to leave the newly established French Departments Bas Rhine and Haut Rhin, losing all their private possessions. When Schmiedeberg stood before the railway station waiting for his transport back to Germany, Albert Schweitzer saw him there with a wrapped package under his arm. Asking whether he could be of help to transfer his furniture to Germany, Schmiedeberg only asked him to take the package and try to safely send it to his new address in Baden-Baden. It was the manuscript of his last paper, and he feared that it might be confiscated before entering the train. Schweitzer took it and later transferred it safely to Schmiedeberg, since then living in Baden-Baden. Oswald Schmiedeberg died on 12 July 1921 in Baden-Baden.

In recognition of Schmiedeberg, the German Society of Experimental and Clinical Pharmacology and Toxicology since 1956 dedicates the Oswald Schmiedeberg Medal to scientists for their extraordinary contributions to pharmacology, clinical pharmacology, and toxicology.

## Data Availability

No datasets were generated or analyzed during the current study.

## Footnotes

1 I thank Giovanni Gori, former editor of Regulatory Toxicology and Pharmacology, for assistance to appropriately translate the original German text into English.

## References

[CR1] Bunge G, Schmiedeberg O (1877) Über die Bildung der Hippursäure. Arch Exp Path Pharmak 6:233–255

[CR2] Heubner W (1956) Erinnerungen an Oswald Schmiedeberg. Studienwerk der Freien Universität Berlin E.V. Mitteilungen Nr. 1

[CR3] Holmstedt B, Liljestrand G (eds) (1963) Readings in Pharmacology. Pergamon Press Ltd, Oxford

[CR4] Koch-Weser J, Schechter PJ (1978) Schmiedeberg in Strassburg 1872–1918. The making of modern pharmacology. Life Sci 22:1361–1372 351320 10.1016/0024-3205(78)90099-1

[CR5] Meyer HH (1922) Schmiedebergs Werk. Arch Exp Path Pharmak 92:1–17

[CR6] Muscholl E (1995) The evolution of experimental pharmacology as a biological science: the pioneering work of Buchheim and Schmiedeberg. Brit J Pharmacol 116:2155–2159 8564242 10.1111/j.1476-5381.1995.tb15047.x PMC1908990

[CR7] Philippou A, Seifert R (2022) History of pharmacology: 2 – The Institute of Pharmacology of the University of Strasbourg: genealogy and biographies. Naunyn-Schmiedeberg’s Arch Pharmacol 361:19–33 10.1007/s00210-022-02336-x 36520164

[CR8] Pohar M, Hansson N (2020) The “Nobel Population” in pharmacology: Nobel Prize laureates, nominees and nominators 1901–1953 with a focus on B. Naunyn and O. Schmiedeberg. Naunyn-Schmeideberg’s Arch Pharmacol 393:1173–1185 10.1007/s00210-019-01807-y 31953675

[CR9] Schmiedeberg O (1870) Untersuchung über einige Giftwirkungen am Froschherzen. In: Berichte über die Verhandlungen der Königlich-Sächsischen Gesellschaft der Wissenschaften zu Leipzig, Mathematisch-Physische Klasse 22:130-141

[CR10] Schmiedeberg O (1872) Über die Innervationsverhältnisse des Hundeherzens. Arbeiten aus der Physiologie: Anstalt zu Leipzig

[CR11] Schmiedeberg O (1886) Über die pharmakologischen Wirkungen und die therapeutische Anwendung einiger Carbaminsäureester. Arch Exp Path Pharmak 20:203–216

[CR12] Schmiedeberg O, Meyer HH (1879) Über Stoffwechselprodukte nach Campherfütterung. Z Physiol Chem 3:422–450

[CR13] Schmiedeberg O (1906) Grundriß der Pharmakologie in Bezug auf Arzneimittellehre und Toxikologie. 5. Edition, Vogel, Leipzig (Digital edition)

[CR14] Schmiedeberg O (1912) Arzneimittel und Genußmittel. Vogel, Leipzig (Digital edition)

[CR15] Schmiedeberg O, Koppe R (1869) Das Muscarin, das giftige Alkaloid des Fliegenpilzes (Agaricus muscarius L.), seine Darstellung, chemischen Eigenschaften, physiologischen Wirkungen, toxicologische Bedeutung und sein Verhältniss zur Pilzvergiftung im allgemeinen. FCW Vogel, Leipzig

